# Preoperative Prediction of Chronic Post-surgical Pain Risk After Total Knee Arthroplasty: Insights From a Single-Center Case-Control Study

**DOI:** 10.7759/cureus.93815

**Published:** 2025-10-04

**Authors:** Bhavesh Santani, Shubhabrata De, Diego Vergara-Jalandoni, Abhina George, Venancio Manipol, Ulfat Sardar, Rakshya Upreti, Ashwin Unnithan

**Affiliations:** 1 General Surgery, Ashford and St. Peter's Hospitals National Health Service (NHS) Foundation Trust, Chertsey, GBR; 2 Internal Medicine, Royal Surrey County Hospital, Guildford, GBR; 3 Trauma and Orthopaedics, Ashford and St. Peter's Hospitals National Health Service (NHS) Foundation Trust, Chertsey, GBR; 4 Internal Medicine, Ashford and St. Peter's Hospitals National Health Service (NHS) Foundation Trust, Chertsey, GBR; 5 Urology, Ashford and St. Peter's Hospitals National Health Service (NHS) Foundation Trust, Chertsey, GBR; 6 Obstetrics and Gynecology, Ashford and St. Peter's Hospitals National Health Service (NHS) Foundation Trust, Chertsey, GBR

**Keywords:** chronic pain, chronic post-surgical pain, knee pain, pain score, post tkr

## Abstract

Background

Chronic post-surgical pain (CPSP) is a common and distressing complication following total knee arthroplasty (TKA). Despite advancements, CPSP remains a significant challenge, necessitating the development of a predictive model to identify at-risk patients.

Materials and methods

This level III case-control study analyzed 869 patients who underwent TKA between 2019 and 2021 at a single center. Data on demographics, comorbidities, mental health conditions, pre-existing pain, opioid use, substance abuse, and surgical factors were collected. Univariate and multivariate logistic regression analyses were conducted to identify significant preoperative risk factors. Independent predictors from multivariate analysis were assigned weighted scores proportional to their odds ratios, which were then adjusted according to previous literature and investigator consensus to create a clinically usable tool. Receiver operating characteristic (ROC) curve analysis was performed, and cutoffs were identified to optimize sensitivity and specificity.

Results

Out of 869 patients who underwent TKA, 15.7% (n = 136) developed CPSP. Out of these 136 patients, 22% (29) were referred to specialist pain management clinics. Univariate analysis identified seven significant predictors: diabetes mellitus, coronary artery disease (CAD), heart failure, mental health conditions, pre-existing pain, and preoperative opioid use. The multivariate analysis excluded heart failure (p = 0.098). The strongest association was found with CAD (OR = 2.98). A scoring system was developed, and an ROC analysis yielded an area under the curve of 0.616 (95% CI: 0.562-0.671, p = 0.001).

Conclusions

Identifying preoperative risk factors and using this score as a predictive model, pending further prospective validation, can help stratify patients and enable targeted interventions, potentially minimizing the impact and financial burden of CPSP after TKA.

## Introduction

The International Classification of Diseases, 11th Revision, defines chronic post-surgical pain (CPSP) as "…pain developing or increasing in intensity after a surgical procedure or a tissue injury (involving any trauma, including burns) and persisting beyond the healing process, i.e., at least three months after surgery or tissue trauma [1]." In 2017, the International Association for the Study of Pain emphasized the critical need to identify surgical patients at risk of developing persistent pain to optimize care [2]. Despite advancements in surgical techniques and postoperative care, approximately 20% of patients still experience chronic pain after total knee arthroplasty (TKA) [3]. A systematic review of prospective studies found that 34% of patients reported unfavorable pain outcomes between three months and five years post-TKA [4]. Orthopedic surgery aimed at alleviating pain has been associated with an almost threefold risk of moderate to severe chronic pain compared to other surgical procedures [5]. The prevalence of CPSP remains a significant challenge, necessitating a comprehensive approach to identifying and mitigating risk factors [6].

CPSP after TKA is a complex condition influenced by multiple biological, surgical, and psychological factors [3]. Biological factors have long-term implications in the development of osteoarthritis and complex regional pain syndrome, as well as in inflammation, infection, and localized nerve injury [7-12]. Psychosocial factors such as depression and poor social support have been linked to worse pain outcomes and greater dissatisfaction rates in patients with chronic pain after TKA [2]. Patients with preoperative psychological distress, including pain catastrophizing, high mental distress, and psychological disorders such as somatization dysfunction, anxiety, and depression, are more likely to experience poor outcomes after TKA compared to those without psychological distress [13,14]. Preoperative opioid use for pain relief is another significant factor, with studies indicating a fourfold increase in CPSP in patients who used opioids preoperatively compared to those with reduced preoperative opioid use [1].

Given the various risk factors for CPSP, there is a need for a predictive model that integrates these factors to provide a practical tool for preoperative assessment [15]. The primary objective of this study was to identify patient-related risk factors associated with CPSP after TKA and to utilize these risk factors in developing a clinically applicable scoring system for risk stratification. The secondary objective of this study was to calculate the prevalence of CPSP in our cohort. The study also lays the foundation for future studies to prospectively validate the predictive score and clinical application, enabling targeted interventions to reduce the impact of CPSP.

## Materials and methods

This was a level III case-control study conducted at a 575-bed public district general hospital (secondary care) to identify preoperative risk factors associated with CPSP following TKA and develop a predictive score based on these factors. This study included patients of all age groups and all genders who underwent TKA at our center between 2019 and 2021 and had at least three months of follow-up. Patients who underwent revision TKA and those who were lost to follow-up within three months post-surgery were excluded.

Ethical approval by an institutional review board was not required for this study, as it involved retrospective analysis of anonymized data from routine clinical care without direct patient involvement or intervention. No a priori sample size calculation was performed due to the retrospective design. All consecutive patients meeting the inclusion criteria during the study period were included to maximize available data and minimize selection bias.

Data collection was performed through a comprehensive review of electronic medical records. The variables extracted included demographics, comorbidities (assessed primarily using components of the Charlson Comorbidity Index), mental health conditions, pre‑existing pain disorders and opioid use, history of substance abuse (smoking and alcohol consumption), and functional status. We obtained follow-up data from electronic patient records to identify patients experiencing chronic pain beyond the postoperative period, as well as those referred to specialist pain management clinics.

Cases were defined as patients experiencing CPSP after TKA, defined according to ICD-11 as pain persisting beyond three months postoperatively, and documented in follow-up history. Controls were defined as patients undergoing TKA in the same period who did not report persistent pain beyond three months.

Data were processed using Microsoft Excel version 2401 (Microsoft Corp., Redmond, WA, USA), and data analyses, including receiver operating characteristic (ROC) curve analysis, were conducted using SPSS Statistics version 27.0 (IBM Corp. Released 2020. IBM SPSS Statistics for Windows, Version 27.0. Armonk, NY: IBM Corp.).

The primary outcome was the presence or absence of CPSP at ≥3 months post‑TKA, treated as a binary variable. Univariate and multivariate logistic regression analyses were employed to identify significant preoperative factors associated with chronic pain. Statistical significance was defined as p < 0.05 for both models. Univariate analysis was used to evaluate each preoperative factor independently, and the variables identified as significant were entered into a multivariate logistic regression model to identify independent predictors. Odds ratios (ORs) with 95% confidence intervals (CIs) were reported. Model calibration was assessed using the Hosmer-Lemeshow goodness‑of‑fit test, and overall explanatory power was summarized with the Nagelkerke R² statistic.

A predictive score was developed based on significant preoperative factors identified from the multivariate regression model. Points were given to each variable based on the relative magnitude of its OR, with higher ORs generally receiving greater weight. Points were then pragmatically adjusted through investigator consensus, based on previous literature and clinical plausibility, to maintain balance between the predictors. ROC curve analysis was performed to evaluate discriminative ability. Permutation testing was used to refine allocations to optimize sensitivity and specificity.

## Results

A total of 920 patients who underwent TKA were identified. Fifty-one patients were excluded because they had undergone revision surgery or were lost to follow-up within the first three months. Therefore, this study included 869 patients. Among these, 57% (n = 495) of the patients were female, and the most significant proportion of patients was in the 70-79 age bracket, which accounted for 37% (n = 329). We found that 15.7% (n = 136) of the patients experienced CPSP after TKA. Twenty-two percent (n = 29) of these patients required referral to specialist pain management clinics to address their CPSP.

Univariate analysis identified seven significant risk factors for CPSP: diabetes mellitus, coronary artery disease (CAD), heart failure, mental health conditions, pre-existing pain conditions, and preoperative use of opioids (Table 1).

**Table 1:** Details of chronic pain incidence in patient with different risk factors with p-value CPSP: chronic post-surgical pain, BMI: body mass index, CVA: cerebrovascular accident (stroke), CAD: coronary artery disease, PVD: peripheral vascular disease, CHF: congestive heart failure, COPD: chronic obstructive pulmonary disease, CKD: chronic kidney disease

Variable		Total		CPSP present		CPSP absent		p-value
		N	%	N	%	N	%	
Sex	F	495	57.0%	80	58.8%	415	56.6%	0.633
	M	374	43.0%	56	41.2%	318	43.4%	
Age group	30-39	1	0.1%	0	0.0%	1	0.1%	0.797
	40-49	15	1.7%	3	2.2%	12	1.6%	
	50-59	118	13.6%	24	17.6%	94	12.8%	
	60-69	271	31.2%	38	27.9%	233	31.8%	
	70-79	329	37.9%	49	36.0%	280	38.2%	
	80-89	129	14.8%	21	15.4%	108	14.7%	
	90-99	6	0.7%	1	0.7%	5	0.7%	
BMI category	Normal	98	11.3%	15	11.0%	83	11.3%	0.481
	Obese I	251	28.9%	46	33.8%	205	28.0%	
	Obese II	142	16.3%	23	16.9%	119	16.2%	
	Obese III	69	7.9%	9	6.6%	60	8.2%	
	Overweight	284	32.7%	42	30.9%	242	33.0%	
	Underweight	25	2.9%	1	0.7%	24	3.3%	
CVA	Yes	36	4.1%	8	5.9%	28	3.8%	0.268
	No	833	95.9%	128	94.1%	705	96.2%	
CAD	Yes	47	5.4%	17	12.5%	30	4.1%	<0.001
	No	822	94.6%	119	87.5%	703	95.9%	
PVD	Yes	13	1.5%	2	1.5%	11	1.5%	0.979
	No	856	98.5%	134	98.5%	722	98.5%	
Dementia	Yes	3	0.3%	1	0.7%	2	0.3%	0.398
	No	866	99.7%	135	99.3%	731	99.7%	
CHF	Yes	15	1.7%	6	4.4%	9	1.2%	0.009
	No	854	98.3%	130	95.6%	724	98.8%	
COPD	Yes	30	3.5%	6	4.4%	24	3.3%	0.505
	No	839	96.5%	130	95.6%	709	96.7%	
Connective tissue disorders	Yes	38	4.4%	3	2.2%	35	4.8%	0.178
	No	831	95.6%	133	97.8%	698	95.2%	
CKD	Yes	38	4.4%	7	5.1%	31	4.2%	0.631
	No	831	95.6%	129	94.9%	702	95.8%	
Diabetes	Yes	129	14.8%	30	22.1%	99	13.5%	0.01
	No	740	85.2%	106	77.9%	634	86.5%	
Mental health conditions	Yes	133	15.3%	33	24.3%	100	13.6%	0.002
	No	736	84.7%	103	75.7%	633	86.4%	
Pain problems	Yes	80	9.2%	22	16.2%	58	7.9%	0.002
	No	789	90.8%	114	83.8%	675	92.1%	
Smoker	Yes	112	12.9%	28	20.6%	84	11.5%	0.004
	No	757	87.1%	108	79.4%	649	88.5%	
Preoperative opioid use	Yes	150	17.3%	35	25.7%	115	15.7%	0.004
	No	719	82.7%	101	74.3%	618	84.3%	

The Kendall tau-b correlation coefficient was calculated for all risk factors, revealing a significant correlation between mental health conditions and pre-existing pain conditions, with a correlation coefficient of 0.357.

The identified significant risk factors (diabetes mellitus, CAD, heart failure, mental health conditions, pre-existing pain conditions, and preoperative use of opioids) were entered into a multivariate binary logistic regression model. Table 2 presents the results of the logistic regression analysis.

**Table 2:** Outcome of multivariate binary logistic regression model OR: odds ratio, CI: confidence interval

Risk factors	p-value	OR	95% CI for OR	
			Lower	Upper
Preoperative opioid use	0.049	1.479	0.928	2.356
Smoker	0.042	1.652	1.000	2.727
Mental health conditions	0.050	1.623	1.003	2.626
Diabetes mellitus	0.018	1.606	0.996	2.590
Coronary artery disease	0.001	2.980	1.541	5.763
Pain problems	0.042	1.753	0.991	3.102
Congestive heart failure	0.098	2.588	0.838	7.989

The Nagelkerke R² for the model is 0.08. The Hosmer-Lemeshow test for goodness of fit indicated a satisfactory fit (p = 0.897). Logistic regression results revealed that heart failure was not a significant predictor (p = 0.098); therefore, it was excluded from the final model.

The final model involved assigning weighting to each risk factor based on its OR, previous studies, and consensus among the investigators, as detailed in the methods section. Table 3 presents the resulting mental health, epicardial-CAD, preoperative opioid use, pain conditions, diabetes mellitus, and smoking (MEOPDS) scoring system with a total score of 9.

**Table 3:** Scores allotted to different components of the MEOPDS score MEOPDS: mental health, epicardial-coronary artery disease, preoperative opioid use, pain conditions, diabetes mellitus, and smoking

S. no.	Risk factor	Score allotted
1	Mental health problems	2
2	Coronary artery disease	2.5
3	Pain conditions	2
4	Preoperative opioid use	0.5
5	Diabetes mellitus	1
6	Smoking	1

**Figure 1:**
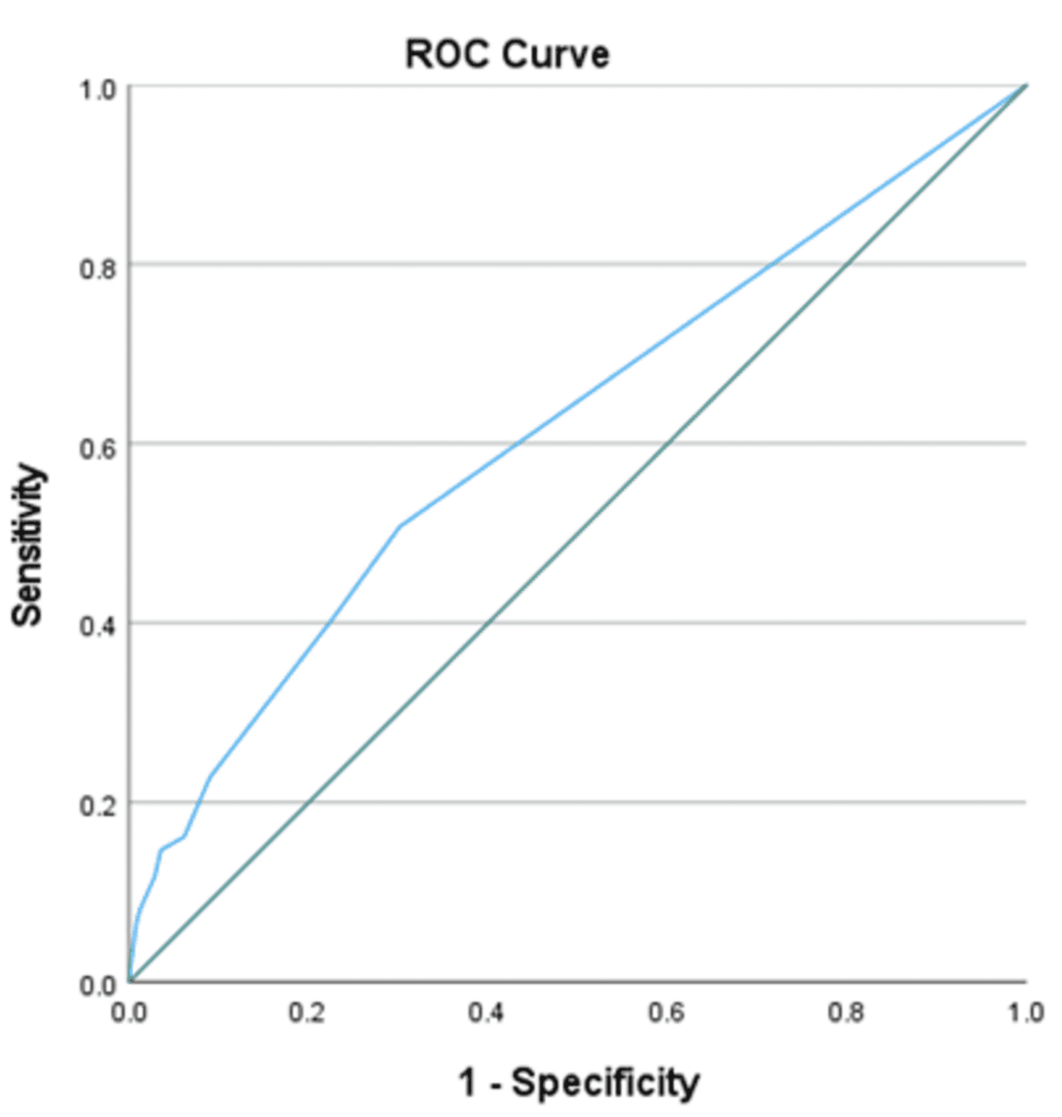
ROC analysis for the predictive model Diagonal segments are produced by ties. ROC: receiver operating characteristic

ROC curve analysis was conducted for the predictive model, yielding an area under the curve (AUC) of 0.616 (95% CI: 0.562-0.671, p = 0.001), as shown in Figure 1.

A cutoff score of 3.5 was determined by examining the curve coordinates, which provided the optimal balance of sensitivity and specificity. This score had a sensitivity of 55.9%, a specificity of 63.6%, a positive predictive value of 22.15%, and a negative predictive value of 88.6%. This scoring system stratifies patients into high- and low-risk categories for the development of CPSP following TKR, with a score of ≥3.5 indicating a high risk for CPSP.

## Discussion

CPSP is a frequent and distressing complication of surgery, leading to functional limitations and psychological distress in patients, while casting doubts about the success of the surgery. Additionally, CPSP increases the overall burden on the healthcare industry when the costs of unfavorable surgeries, pain management, and other related expenses are included. While complete prevention remains elusive, this study aimed to identify statistically significant preoperative risk factors for CPSP in patients undergoing TKA and implement suitable preventive measures preoperatively.

This study examined a wide variety of risk factors, including psychological conditions, age, sex, genetics, body mass index, comorbidities, addiction, and preoperative opioid use. Univariate analysis identified a positive association with seven preoperative factors with significant p-values: diabetes mellitus, CAD, smoking, heart failure, mental health conditions, pre-existing pain conditions, and preoperative use of opioids. Although univariate analyses offer specific information about each variable of interest, they do not account for other confounding factors or correlations among the risk factors, which could limit their value. However, they provide more direct information on each variable.

Among the remaining six significant risk factors, the strongest association (OR = 2.98) was found between a history of CAD and CPSP. Notably, previous studies have not documented this association with CAD, and the lack of a significant association with other vascular problems, such as peripheral vascular disease, is puzzling. This discrepancy highlights the need for further research in this area. In the multivariate models, heart failure was found to be insignificant (p = 0.098); therefore, it was excluded from the final model. Since patients with heart failure mostly have a history of previous cardiac ischemic events, this may have been a confounding factor, as there was a significant correlation with CAD.

These findings support previous systematic reviews and meta-analyses that have consistently identified mental health conditions and pre-existing pain problems as independent predictors of persistent pain after TKA [16]. Interestingly, this study did not find that female sex or younger age at surgery were significant risk factors for CPSP, contrary to frequent identifications in other studies [17,18]. The higher likelihood of comorbidities in older patients likely confounds the potential increased risk associated with younger age at surgery [19,20]. Diabetes mellitus and preoperative opioid use have also been shown to increase the absolute risk of CPSP, and this study confirms these findings [21,22].

A scoring system was developed based on the six identified significant factors, weighted according to their odds ratios, previous study results, and consensus among investigators. Given that the predictive model demonstrated only modest discriminative ability, with an AUC of 0.616, and considering the retrospective, single-center nature of this study, further research and prospective/external validation are necessary to refine and enhance its accuracy.

We acknowledge the limitations of our study. Currently, the predictive ability of the score is limited, as the AUC is <0.7. Further larger-scale studies will be required to refine the score and also for validation.

Since the study was conducted in a single center, this may limit its external validity, as institutional practices and patient cohorts may differ elsewhere. Another limitation of the study is that the data collection was done from electronic health records. This may have led to some data being omitted due to improper/missing documentation, although investigators made every effort to avoid missing any variables.

Data about some important predictors of CPSP, as demonstrated in previous studies, such as pain catastrophizing and preoperative pain severity scores, were not available. Additionally, CPSP was treated as a binary outcome, which simplifies analysis but may overlook the multidimensional nature of chronic pain.

No a priori sample size calculation was performed; instead, all consecutive eligible patients were included to maximize available data and minimize selection bias. We acknowledge that the absence of a planned sample size may increase the possibility of reduced statistical power and wider CIs for rarer predictors.

Data on surgical technique and other intraoperative variables, such as implant type, approach, and soft tissue balancing, were not captured, as the main focus of the study was on preoperative variables. These factors may nonetheless influence the development of CPSP [10]. Stratification by surgical variables in future prospective studies could reduce potential confounding with preoperative factors.

The use of robotic-assisted TKA has demonstrated increasing adoption in current orthopedic practice. At our institution, robotic TKA procedures were initiated in 2022 with the implementation of generation II robotic systems. This provides opportunities for further studies to be directed at comparing chronic pain outcomes in surgeries performed with traditional techniques versus robotic techniques and applying the score to assess differences in the predictive ability of the score.

While the current model has limited discriminative capacity, its preoperative risk stratification could guide the targeted use of robotic TKA systems, which have been shown to improve bony resection accuracy and soft-tissue balancing, factors implicated in persistent post-TKA pain [23].

Previous research has shown that patient satisfaction and clinical outcomes are key topics of recent studies examining robotic TKA systems [24]. It would be interesting to see if combining such predictive models with robotic TKA systems would lead to better outcomes for patients identified at higher risk of developing chronic pain.

We are looking to implement a scoring system at our center to stratify patients into high- and low-risk groups. Although this is a retrospective study, further external validation of the score may enable targeted preoperative counseling to be offered to patients in the high-risk group, potentially leading to improved patient satisfaction [15].

Finally, the financial burden of CPSP post-TKA from this patient cohort was calculated based on the cost of management interventions derived from the National Cost Collection of NHS costs in NHS trusts and NHS Foundation trusts for the financial years 2019-2020 [25]. The cost of surgery, additional appointments, and investigations performed for patients who developed CPSP amounted to £1,298,000. The implementation of a validated scoring system in NHS trusts nationwide could potentially lead to improved patient satisfaction rates and may even help reduce the cost burden.

## Conclusions

CPSP post-TKA remains a significant challenge despite advancements in surgical techniques and postoperative care. Identifying preoperative risk factors and developing the MEOPDS score as a predictive model can help stratify patients based on their risk, enabling targeted interventions such as specialized pain management counseling to reduce the incidence of CPSP. While the MEOPDS score provides an initial framework for risk stratification, its predictive performance in this dataset was modest. Validation with larger multicenter datasets is required to refine the model, improve its discriminatory ability, and confirm its clinical utility. This approach has the potential to minimize the impact of CPSP and reduce the financial burden on healthcare systems; however, it still requires further research to strengthen the predictive capabilities of such models.
